# The contributions of ankle, knee and hip joint work to individual leg work change during uphill and downhill walking over a range of speeds

**DOI:** 10.1098/rsos.180550

**Published:** 2018-08-29

**Authors:** Jana R. Montgomery, Alena M. Grabowski

**Affiliations:** 1 Integrative Physiology, University of Colorado Boulder, Boulder, CO, USA; 2 VA Eastern Colorado Health Care System, Denver, CO, USA

**Keywords:** gait, biomechanics, prostheses, amputation, slope

## Abstract

The muscles surrounding the ankle, knee and hip joints provide 42, 16 and 42%, respectively, of the total leg positive power required to walk on level ground at various speeds. However, each joint's contribution to leg work when walking up/downhill at a range of speeds is not known. Determining each biological joint's contribution to leg work over a range of speeds and slopes can inform the design of biomimetic assistive devices (i.e. prostheses). Twenty healthy adults walked 1.00, 1.25 and 1.50 m s^−1^ on 0°, ±3°, ±6° and ±9° while we collected kinematic and kinetic data. We calculated sagittal plane joint work and individual leg work over the entire stance phase. The ratio of ankle joint to total individual leg positive work (summed ankle, knee and hip joint work) did not change (0.42) with speed or slope, but the ratio of ankle joint to individual leg negative work was 0.38 at −9°, 0.42 at 0° and 0.27 at +9° across all speeds. The ratio of ankle joint to total individual leg negative work was 0.41 at 1.00 m s^−1^ and 0.32 at 1.50 m s^−1^ across all slopes. The ratio of knee joint to total individual positive leg work (0.22) did not change with speed or slope. The ratio of knee joint to total individual leg negative work was 0.39 at 1.00 m s^−1^ and 0.45 at 1.50 m s^−1^ across all slopes. The ratio of hip joint to total individual leg positive work did not change with speed but was 0.34 at −9°, 0.33 at 0° and 0.37 at +9° across all speeds. The ratio of hip joint to total individual leg negative work was 0.21 at 1.00 m s^−1^, and 0.24 at 1.50 m s^−1^ across all slopes and 0.17 at −9°, 0.19 at 0° and 0.29 at +9° across all speeds. The ankle significantly contributes to walking on slopes and this contribution changes during sloped compared with level-ground walking, thus assistive devices that provide biomimetic ankle function must adapt to accommodate walking at different speeds and slopes; whereas assistive biomimetic devices for the knee only need to adapt at different speeds.

## 1. Introduction

The net mechanical work done on the centre of mass (COM) during level-ground walking is presumably near zero over a stride. However, to enable level-ground walking, the muscles surrounding the ankle, knee and hip joints produce net positive mechanical work. The biomechanics of the single support phase of walking have been described using an inverted pendulum model, where the body's COM is represented by a point mass, and the leg by a rigid, massless strut [1–4]. This model suggests that minimal external mechanical work is necessary to sustain steady-speed level-ground walking during single support because there is a constant phasic exchange of potential and kinetic energy [1,3]. Thus, during a single stride at a steady speed on level ground, the net external mechanical work done on the body's COM would presumably be near zero [2,3]. However, multiple studies have found that during level-ground walking at a constant speed, the net mechanical work done on the COM per step is approximately 0.22 J kg^−1^ [5,6] and per stride is up to 4.0 J (0.05 J kg^−1^) [7]. These findings are probably due to mechanical work required during the step-to-step transition phase. During the step-to-step transition phase of level-ground walking, the muscles of each leg must perform both negative and positive work on the COM simultaneously [8]. The leading leg absorbs mechanical work as it slows the downward and forward velocity of the COM, while the trailing leg simultaneously produces mechanical work to redirect and reaccelerate the COM upward and forward [9–11]. Moreover, individual leg step-to-step transition work constitutes the majority of the external mechanical work done during walking [1,2,4,9,12,13]. Further, the mechanical work performed to redirect and accelerate the COM requires approximately 45% of the net metabolic power for level-ground walking [14], whereas supporting body weight (primarily during single support) comprises approximately 28% of the net metabolic power [14]. Thus, powered knee and ankle–foot prostheses have been developed to replicate normative walking mechanics by providing stance phase power primarily during the step-to-step transition [15].

Some previous studies have used the combined limbs method to account for the total external mechanical work done by both legs on the COM during the single and double support phases of walking [1,16]. Using this method, a previous study suggested that the muscles surrounding the ankle joint contribute approximately 80% of the overall mechanical leg work required to walk on level ground [17]. However, the simultaneous positive and negative work done by the leading and trailing legs during the step-to-step transition are mathematically cancelled out when using the combined limbs method. By contrast, the individual limbs method, which accounts for the work done by each individual leg during the step-to-step transition, provides a 31% higher estimate of the COM work [10]. Further, the muscles surrounding the ankle provide approximately 42%, the muscles surrounding the hip provide approximately 42% and the muscles surrounding the knee provide approximately 16% of the total positive individual leg power during level-ground walking over a range of speeds when using inverse dynamics to calculate joint power and summing all joint positive power to determine individual leg total positive power [18].

When walking at different speeds on level ground, uphill and downhill slopes, individual leg mechanical work during a stride changes [6], and joint work also changes during the stance phase [19]. Further, because leg work changes throughout the stance phase of walking, it is important to consider joint mechanics and each joint's contribution to leg mechanics during the entire stance phase rather than only during single support or the step-to-step transition. For example, Franz *et al*. [6] quantified the mechanical work done by the leading and trailing legs throughout the stance phase and demonstrated that each leg absorbs and generates mechanical work. The leading and trailing legs primarily generate positive work during uphill walking on slopes of +3° to +9°, and primarily absorb negative work when walking downhill on slopes of −3° to −9° throughout the entire stance phase [6]. In other words, walking uphill requires almost no negative leg work (0.14–0.02 J kg^−1^) and walking downhill requires almost no positive leg work (0.05–0.18 J kg^−1^) [6].

Similarly, the power and work generated by the muscles surrounding the ankle, knee and hip joints differ in magnitude when walking uphill or downhill. Lay *et al*. [20] found that the muscles acting at the ankle generate two times greater peak power and the muscles acting at the hip generate seven times greater peak power when walking up a 21.5° slope compared to level ground. By contrast, the muscles acting at the knee absorb 40% more peak negative power when walking downhill at 21.5° compared to level ground [20]. These data indicate that each joint individually adapts to different slopes and performs a different amount of work. Similar to walking uphill, increasing walking speed on level ground requires the legs to produce more positive power and perform more positive work on the COM. However, contrary to uphill walking, increasing walking speed on level ground requires the legs to also absorb more negative work. When walking on level ground, total leg positive power increases with speed, but each joint's contribution to total positive individual leg power remains constant; the ankle contributes 42%, while the knee and hip contribute 16 and 42%, respectively [18]. To better understand the contribution of lower-limb joint work during walking on slopes, Alexander *et al*. determined ankle, knee and hip joint work from walking at 1.1 m s^−1^ on ramps of 0°, ±6°, ±12° and ±18°. In general, they found that the muscles surrounding the ankle joint perform almost three times the amount of positive work at a steep uphill slope of +18° compared to level ground, the muscles surrounding the knee joint absorb almost eight times as much negative work when walking on a downhill slope of −18° compared to level ground and the muscles surrounding the hip joint increase positive work by almost six times when walking on an uphill slope of +18° compared to level ground [19]. While joint and individual leg positive, negative and net work were reported for a range of slopes in Alexander *et al*. [19], subjects walked at one speed on a ramp, which was used to collect data from non-consecutive steps (multiple trials were performed at each inclination). However, each joint's relative contribution to individual leg mechanical positive or negative work or power when walking over a range of speeds on different slopes has not yet been determined.

Similar to sagittal plane joint power, peak ankle, knee and hip joint sagittal plane moments increase during uphill (21°) compared to level-ground walking, whereas peak ankle moment decreases, peak knee extensor moment increases and peak hip moment remains unchanged during downhill (−21°) compared to level-ground walking [21]. Similarly, ankle and hip joint sagittal plane range of motion (ROM) increase while the knee joint sagittal plane ROM remains unchanged during uphill compared to level-ground walking [21]. Conversely, peak knee joint flexion increases, while ankle and hip joint total ROM remain unchanged during downhill compared to level-ground walking [21]. These data suggest that each joint has a different role in successfully navigating uphill and downhill slopes. Further, these biological joint biomechanics data are used to inform the design, development and control of powered assistive devices, such as powered ankle–foot orthoses [22], powered ankle–foot prostheses [23] and powered knee prostheses [15].

A more comprehensive understanding of each joint's kinematics, kinetics and contribution to mechanical work during walking at different speeds and slopes has important implications for the design and control of biomimetic assistive devices such as powered ankle–foot orthoses and prostheses for people that lack normative ankle function, and powered knee prostheses for people with transfemoral amputations. Leg and joint work are commonly calculated as the integral of mechanical power with respect to time, which equals joint angle multiplied by joint moment. Thus, ankle joint moment, ROM and the relationship between these variables, or work loops, are practical measures for understanding ankle function and have been used for powered prosthetic control [17,24–26]. To better replicate biological ankle function, lower-limb prostheses now include an untethered power supply and actuators to produce biomimetic forces and torques about a prosthetic ankle joint during the stance phase of level-ground walking [23,27–29]. The use of a powered ankle–foot prosthesis that permits an ROM similar to and that is capable of generating net positive work equivalent to that of a biological ankle [23,30,31] has resulted in significantly lower metabolic costs for people with a transtibial amputation during level-ground walking over a wide range of speeds compared with the use of a passive-elastic prosthesis, and in equivalent metabolic costs compared to non-amputees [23,27]. Similarly, a powered knee prosthesis that replicates biological knee kinetics and kinematics has normalized the self-selected walking speed of a person with a transfemoral amputation walking over ground [32,33]. Furthermore, a passive ankle exoskeleton that provides plantarflexion assistance has reduced the metabolic cost of level-ground walking at 1.25 m s^−1^ by 7% [34]. While level-ground walking has been the focus of many previous studies and assistive device designs, the ability to vary speed and traverse more complex terrain with varying slopes is important for providing normative function. Thus, a comprehensive analysis of biological joint kinematics and kinetics during walking on varied terrain is needed to improve the design and control of biomimetic powered prostheses, exoskeletons and powered orthoses/assistive devices.

We sought to quantify each biological joint's relative contribution to individual leg work during the entire stance phase of walking across a wide range of slopes and speeds; information that can be used for biomimetic prosthetic and assistive device design. We also sought to quantify biological joint kinematics, work and power across a wide range of uphill and downhill slopes and speeds; information that can be used to inform the control strategies and actuation required for biomimetic prostheses and assistive devices to accommodate slopes. Thus, our overall goal was to provide guidance and insight into the future designs of biomimetic prostheses and orthoses. Based on previous studies [6,19,21,35–37], we anticipated that total positive individual leg and joint work would increase with uphill slopes and hypothesized that: (i) the ratio of positive ankle and hip work to total positive individual leg work would be greater on steeper uphill slopes compared with level-ground walking. Based on previous studies, we expected that total negative individual leg and joint work would increase in magnitude with downhill slopes [37]. We also hypothesized that: (ii) the ratio of negative ankle and hip work to total negative individual leg work would be smaller and the ratio of negative knee work to total negative individual leg work would be larger on steeper downhill slopes compared with level-ground walking. We predicted that ankle, knee and hip joint ROM would not change with speed or downhill slope but would increase with steeper uphill slopes [21]. Finally, we predicted that peak ankle, knee and hip joint moment and peak power would increase (become more positive) on uphill slopes compared with level ground, would decrease (become more negative) on downhill slopes compared to level ground and would increase with speed on all slopes in accordance with previous studies [5,21].

## 2. Methods

### 2.1. Subjects

All subjects gave their written informed consent prior to participating in this study according to a protocol approved by the United States Department of Veteran Affairs' Human Subjects Institutional Review Board and in accordance with the principles expressed in the Declaration of Helsinki. Twenty healthy human subjects with no prior history of lower limb or neurological injury or pathology volunteered (12 M, 8 F, mean age 26.6 years (s.d. 6.18); mean mass 68.7 kg (s.d. 8.4)).

### 2.2. Experimental protocol

We placed reflective markers bilaterally on the malleoli, most posterior point of the heels, first and fifth metatarsal heads, medial and lateral femoral epicondyles, greater trochanters, iliac crests, anterior superior iliac spines and posterior superior iliac spines, and we placed clusters of four markers on each subject's shanks and thighs. Subjects walked on a dual-belt force-measuring treadmill (1000 Hz; Bertec Corp., Columbus, OH, USA) on seven slopes (0°, ±3°, ±6° and ±9°) and at three speeds (1.00, 1.25 and 1.50 m s^−1^) per slope. We simultaneously collected motion and force data for 30 s during each trial (21 trials total) and randomized the order of the trials. Between each trial, we zeroed the treadmill to account for any potential drift in the force transducers. By accounting for drift in the force transducers, we ensured that force magnitude and centre of pressure values were as accurate as possible to yield inverse dynamics calculations.

### 2.3. Kinetics and kinematics

**Figure 1.**
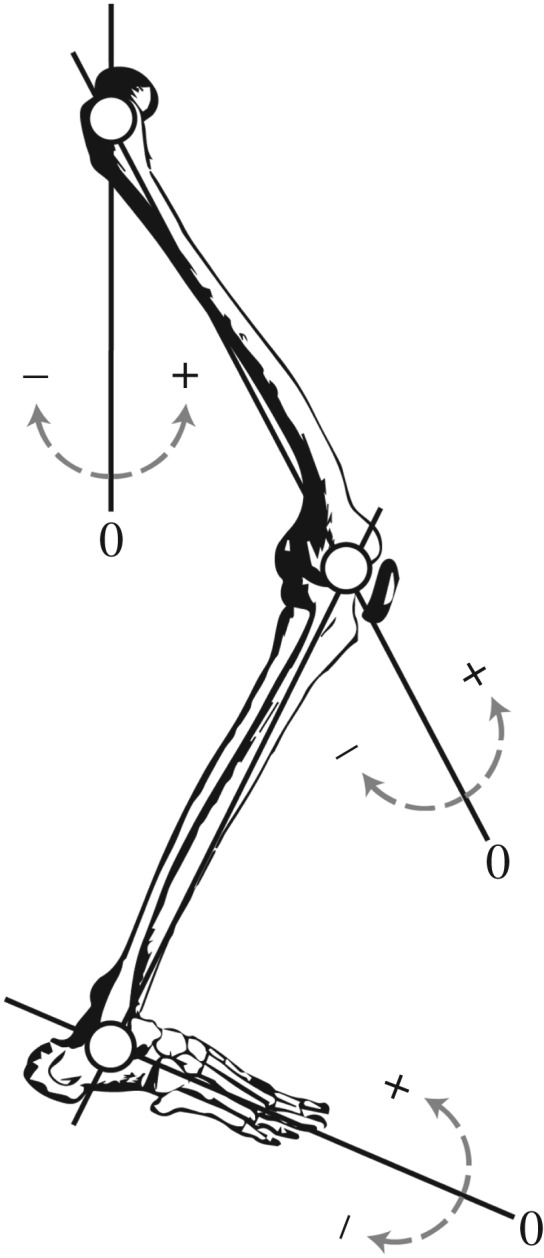
Schematic of segment and corresponding joint angle coordinate systems. Joint centres are represented by white circles. The solid lines indicate 0 degrees, + and − designate positive and negative angles, respectively.

We measured three-dimensional (3D) ground reaction forces (GRFs) from each leg and normalized all data to each subject's mass (*m*). We filtered GRFs with a fourth-order low-pass Butterworth filter and 30 Hz cut-off frequency using a custom software program (Matlab, Mathworks, Natick, MA, USA). We used a 10-camera motion capture system to simultaneously measure 3D kinematics (100 Hz; Vicon Motion Systems; Oxford, UK) and kinetics. Then we filtered the kinematic data with a zero phase shift fourth-order Butterworth low-pass filter and 7 Hz cut-off frequency using Visual3D software (C-Motion, Gaithersburg, MD, USA). We then calculated sagittal plane joint angles, moments and powers (electronic supplementary material, figures S1–S3) based on calculated joint centres (figure 1) and filtered GRFs using Visual3D. We determined ground contact by detecting a 20 N threshold in the vertical GRF using a custom software program (Matlab).

We calculated net sagittal plane joint work (*W*_netJ_) for each leg as the integral of sagittal plane joint power (*P_J_*) from touch-down (td) to toe-off (to) (stance phase) with respect to time (equation (2.1)):

2.1 $$W_{\mathrm{netJ}}=\int_{\mathrm{td}}^{\mathrm{to}}P_{J}\;dt.$$

Positive and negative joint works were then calculated as the integral of sagittal plane joint power during the periods where joint power was positive or negative, respectively, during the stance phase.

To determine the external mechanical work from each individual leg, we summed the joint work calculated via inverse dynamics and integrated the positive and negative portions of joint power. We summed all positive work done by the ankle, knee and hip to determine the total positive work done by the individual leg and summed all negative work done by the ankle, knee and hip to determine the total negative work done by the individual leg. We calculated net work as the sum of total positive and total negative work.

Each joint's contribution to individual leg total positive or negative work was calculated as the ratio of joint work to leg work:

2.2 $$\text{joint's contribution}=\frac{\text{joint work}}{\text{individual leg work}}.$$

### 2.4. Statistics

Prior to choosing a statistical approach to determine the effects of speed, slope and their interaction on individual leg and joint mechanics, we tested for linearity and normality of the data with RStudio statistical software (RStudio, Boston, MA, USA). We determined linearity by visually inspecting residuals and *Q*–*Q* plots in RStudio [38]. Similarly, we determined normality by visually inspecting histograms in RStudio. The data were not linearly related but were normally distributed. Thus, we used two-way repeated-measures ANOVAs (*p* < 0.05) to determine the effects of speed, slope and their interaction on individual leg total positive, total negative and net work, each joint's positive, negative and net work and each joint's contribution to individual leg work. When the ANOVA revealed significance, we used pair-wise independent *t*-tests using a critical *p*-value corrected via the Bonferroni method to compare between speeds and between slopes and level ground. All statistical analyses were done using RStudio.

## 3. Results

### 3.1. Joint total positive, negative and net work

**Figure 2.**
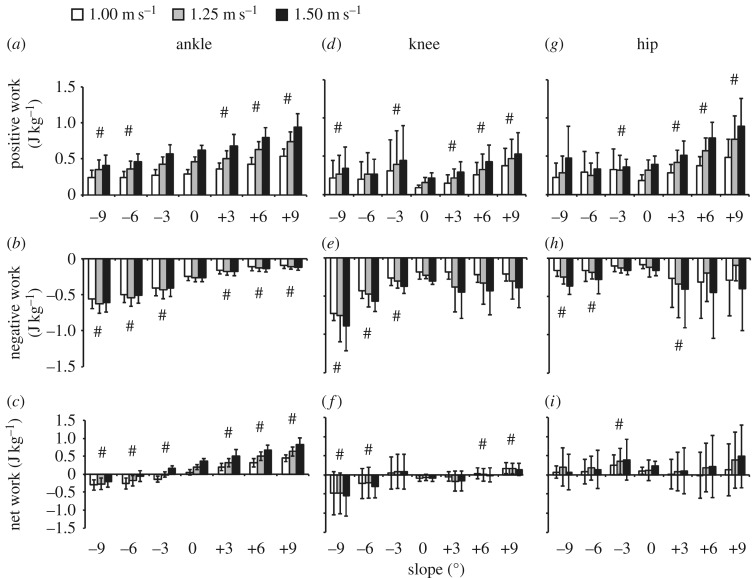
Average (s.d.) (*a*–*c*) ankle, (*d*–*f*) knee and (*g*–*i*) hip joint (*a*,*d*,*g*) total positive, (*b*,*e*,*h*) total negative and (*c*,*f*,*i*) net work over the stance phase for all subjects walking at 1.00 (white), 1.25 (grey), and 1.50 (black) m s^−1^ on slopes of −9° to +9°. # indicates significantly different from level ground. Significant differences between speeds are discussed in the Results section.

We found an effect of speed, slope, and the interaction of speed and slope on positive ankle work (*p* < 0.001, figure 2). Averaged across all slopes, positive ankle work values at all speeds were different from each other (*p* < 0.001, figure 2). Averaged across all speeds, positive ankle work for each slope was different from level ground (*p* < 0.001) except at −3° (*p* = 0.007, Bonferroni-corrected critical *α* = 0.006, figure 2). On level ground, positive ankle work more than doubled from 1.00 to 1.50 m s^−1^ (figure 2). At 1.25 m s^−1^, positive ankle work increased 31% from −9° to level ground (figure 2). Similarly, at 1.25 m s^−1^, positive ankle work increased 61% from level ground to +9° (figure 2).

We found an effect of speed and slope on negative ankle work (*p* < 0.05, figure 2), but there was no effect of the interaction of speed and slope on negative ankle work (*p* = 0.735, figure 2). Averaged across all slopes, negative ankle work at 1.00 m s^−1^ was different from 1.25 and 1.50 m s^−1^ (*p* < 0.001, figure 2). Averaged across all speeds, negative ankle work for each slope was different from level ground (*p* < 0.001, figure 2). On level ground, the magnitude of negative ankle work increased by 8% from 1.00 m s^−1^ to 1.50 m s^−1^ (figure 2). At 1.25 m s^−1^, the magnitude of negative ankle work decreased 58% from −9° to level ground (figure 2). Similarly, at 1.25 m s^−1^, the magnitude of negative ankle work decreased 58% from level ground to +9° slope (figure 2).

We found an effect of speed, slope and the interaction of speed and slope on net ankle work (*p* < 0.001, figure 2). Averaged across all slopes, net ankle work values at each speed were different from each other (*p* < 0.001, figure 2). Averaged across all speeds, net ankle work for each slope was different from level ground (*p* < 0.001, figure 2). On level ground, the magnitude of net ankle work increased sixfold from 1.00 to 1.50 m s^−1^ (figure 2). At 1.25 m s^−1^, net ankle work increased 174% from −9° to level ground (figure 2). Similarly, at 1.25 m s^−1^, net ankle work more than tripled from level ground to +9° (figure 2).

We found an effect of speed and slope on positive knee work (*p* < 0.001, figure 2). There was no effect of the interaction of speed and slope on positive knee work (*p* = 0.875, figure 2). Averaged across all slopes, positive knee work values at each speed were different from each other (*p* < 0.001, figure 2). Averaged across all speeds, positive knee work at each slope was different from level ground (*p* < 0.001) except at −6° (*p* = 0.04, Bonferroni-corrected critical *α* = 0.006, figure 2). On level ground, positive knee work more than doubled from 1.00 to 1.50 m s^−1^ (figure 2). At 1.25 m s^−1^, positive knee work decreased 41% from −9° to level ground (figure 2). Similarly, at 1.25 m s^−1^, positive knee work almost tripled from level ground to +9° (figure 2).

We found an effect of speed and slope on negative knee work (*p* < 0.001, figure 2). There was no effect of the interaction of speed and slope on negative knee work (*p* = 0.708, figure 2). Averaged across all slopes, negative knee work values at each speed were different from each other (*p* < 0.001, figure 2). Averaged across all speeds, negative knee work on each downhill slope was different from level ground (*p* < 0.001, figure 2). On level ground, the magnitude of negative knee work increased 130% from 1.00 to 1.50 m s^−1^ (figure 2). At 1.25 m s^−1^, the magnitude of negative knee work decreased 71% from −9° to level ground (figure 2). At 1.25 m s^−1^, the magnitude of negative knee work increased 41% from level ground to +9° (figure 2).

We found an effect of slope on net knee work (*p* < 0.001, figure 2). We did not find an effect of speed (*p* = 0.346) or the interaction of speed and slope on net knee work (*p* = 0.071, figure 2). Averaged across all speeds, net knee work at each slope was different from level ground (*p* < 0.001) except at −3° and +3° (*p* > 0.02, figure 2). At 1.25 m s^−1^, the magnitude of net knee work decreased 85% from −9° to level ground (figure 2). Similarly, at 1.25 m s^−1^, net knee work increased 328% from level ground to +9° (figure 2).

We found an effect of speed and slope on positive hip work (*p* < 0.001, figure 2). There was no effect of the interaction of speed and slope on positive hip work (*p* = 0.067, figure 2). Averaged across all slopes, positive hip work values at each speed were different from each other (*p* < 0.001, figure 2). Averaged across all speeds, positive hip work at each slope was different from level ground (*p* < 0.001) except at −9° and −6° (*p* > 0.271, figure 2). On level ground, positive hip work more than doubled from 1.00 to 1.50 m s^−1^ (figure 2). At 1.25 m s^−1^, positive hip work increased 14% from −9° to level ground (figure 2). At 1.25 m s^−1^, positive hip work more than doubled from level ground to +9° (figure 2).

We found an effect of speed and slope on negative hip work (*p* < 0.05, figure 2). There was no effect of the interaction of speed and slope on negative hip work (*p* = 0.753, figure 2). Averaged across all slopes, negative hip work values at each speed were different from each other (*p* < 0.001, figure 2). Averaged across all speeds, negative hip work on −9°, −6° and +3° slopes was different from level ground (*p* < 0.001, figure 2). On level ground, the magnitude of negative hip work doubled from 1.00 to 1.50 m s^−1^ (figure 2). At 1.25 m s^−1^, the magnitude of negative hip work decreased 50% from −9° to level ground (figure 2). At 1.25 m s^−1^, the magnitude of negative hip work decreased 17% from level ground to +9° (figure 2).

We found an effect of speed and slope on net hip work (*p* < 0.05, figure 2). There was no effect of the interaction of speed and slope on net hip work (*p* = 0.190, figure 2). Averaged across all slopes, net hip work values at each speed were different from each other (*p* < 0.005, figure 2). Averaged across all speeds, net hip work on the −3° slope was different from level ground (*p* < 0.004, figure 2). On level ground, net hip work more than doubled from 1.00 to 1.50 m s^−1^ (figure 2). At 1.25 m s^−1^, net hip work decreased 43% from −9° to level ground (figure 2). At 1.25 m s^−1^, net hip work more than tripled from level ground to +9° (figure 2).

### 3.2. Individual leg positive, negative and net work

**Figure 3.**
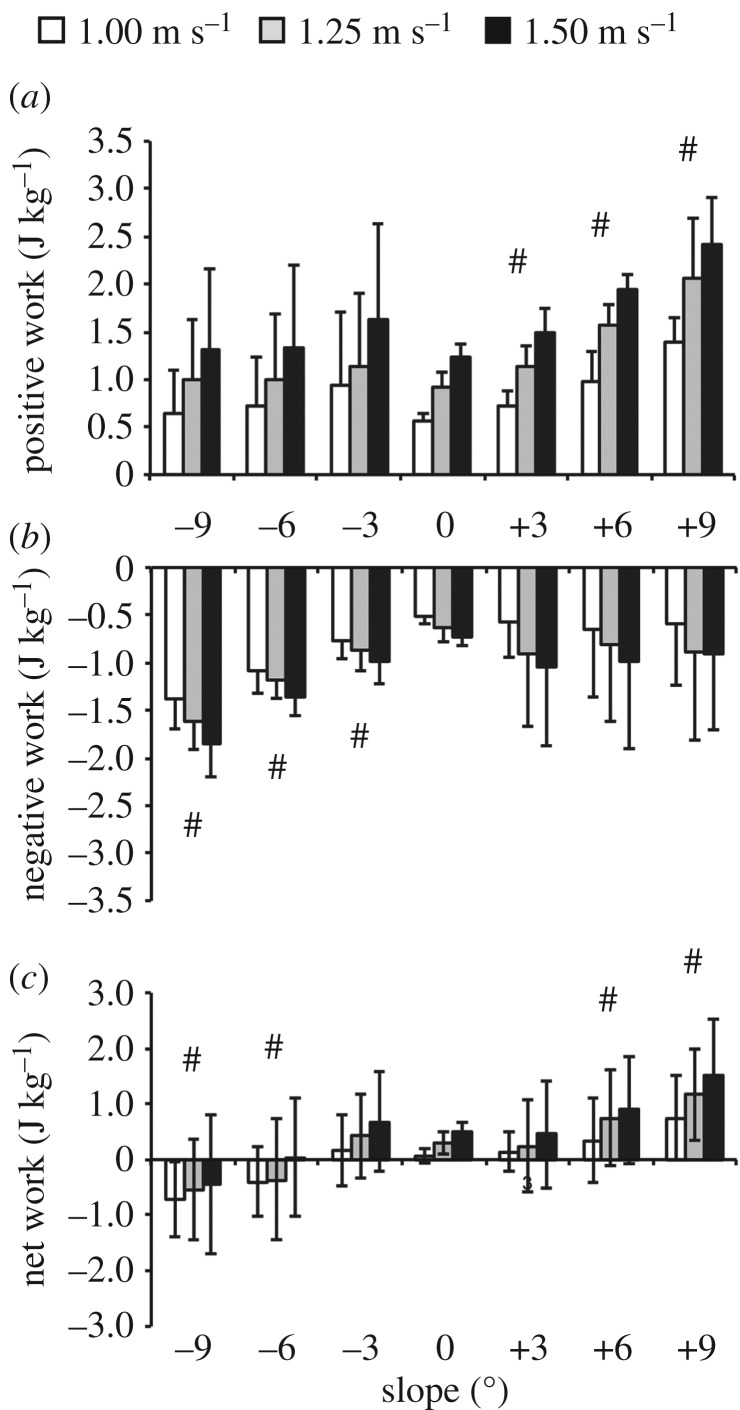
Average (s.d.) individual leg (*a*) total positive, (*b*) total negative and (*c*) net work over the stance phase for all subjects walking at 1.00 (white), 1.25 (grey), and 1.50 (black) m s^−1^ on slopes of −9° to +9°. # indicates significantly different from level ground. Significant differences between speeds are discussed in the Results section.

We found an effect of speed, slope (*p* < 0.001) and their interaction (*p* < 0.05) on positive individual leg work (figure 3). Averaged across all slopes, positive individual leg work values at each speed were different from each other (*p* < 0.001, figure 3). Averaged across all speeds, positive individual leg work on each uphill slope was different from level ground (*p* < 0.001, figure 3). On level ground, positive individual leg work more than doubled from 1.00 to 1.50 m s^−1^ (figure 3). At 1.25 m s^−1^, positive individual leg work decreased 7% from −9° level ground (figure 3). At 1.25 m s^−1^, positive individual leg work more than doubled from level ground to +9° (figure 3).

We found an effect of speed and slope on negative individual leg work (*p* < 0.001, figure 3). There was no effect of the interaction of speed and slope on negative individual leg work (*p* = 0.968, figure 3). Averaged across all slopes, negative individual leg work values at each speed were different from each other (*p* < 0.001, figure 3). Averaged across all speeds, negative individual leg work on each downhill slope was different from level ground (*p* < 0.001, figure 3). On level ground, the magnitude of negative individual leg work increased 41% from 1.00 to 1.50 m s^−1^ (figure 3). At 1.25 m s^−1^, the magnitude of negative individual leg work decreased 62% from −9° to level ground (figure 3).

We found an effect of speed and slope on net individual leg work (*p* < 0.001, figure 3). There was no effect of the interaction of speed and slope on net individual leg work (*p* = 0.122, figure 3). Averaged across all slopes, net individual leg work values at each speed were different from each other (*p* < 0.001, figure 3). Averaged across all speeds, net individual leg work on each slope was different from level ground (*p* < 0.001) except at −3° and +3° (*p* > 0.518, figure 3). On level ground, net individual leg work magnitude increased over sevenfold from 1.00 to 1.50 m s^−1^ (figure 3). At 1.25 m s^−1^, net individual leg work magnitude increased 157% from −9° to level ground (figure 3). At 1.25 m s^−1^, net individual leg work increased 3.7-fold from level ground to +9° (figure 3).

### 3.3. Each joint's contribution to individual leg positive and negative work

**Figure 4.**
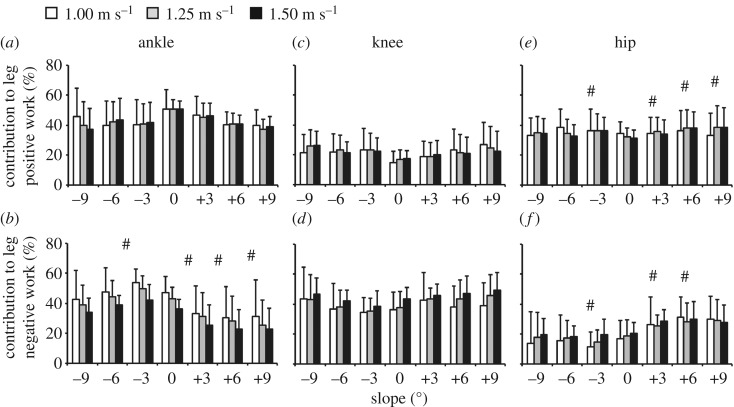
Average (s.d.) contribution (%) of the (*a*,*b*) ankle joint, (*c*,*d*) knee joint and (*e*,*f*) hip joint to (*a*,*c*,*e*) total positive and (*b*,*d*,*f*) total negative individual leg work over the stance phase for all subjects walking at 1.00 (white), 1.25 (grey), and 1.50 (black) m s^−1^ on slopes of −9° to +9°. # indicates significantly difference from level ground. Significant differences between speeds are discussed in the Results section.

We did not find an effect of speed, slope or their interaction on the ratio of ankle positive to total individual leg positive work (*p* > 0.261, figure 4). However, there was an effect of speed and slope (*p* < 0.001), but not the interaction of speed and slope (*p* = 0.979) on the ratio of ankle negative to total individual leg negative work (figure 4). Averaged across all slopes, the ratios of ankle negative to total individual leg negative work at each speed were different from each other (*p* < 0.001, figure 4). Averaged across all speeds, the ratios of ankle negative to total individual leg negative work at each slope were different from level ground (*p* < 0.001) except at −9° and −6° (*p* > 0.064, figure 4). On level ground, the ratio of ankle positive to total individual leg positive work was 0.51 (figure 4). Averaged across all slopes, at 1.25 m s^−1^, the ratio of ankle positive to total individual leg positive work was 0.42 (figure 4). On level ground, the ratio of ankle negative to total individual leg negative work decreased 23% from 1.00 to 1.50 m s^−1^ (figure 4). At 1.25 m s^−1^, the ratio of ankle negative to total individual leg negative work increased 10% from −9° to level ground (figure 4). At 1.25 m s^−1^, the ratio of ankle negative to total individual leg negative work decreased 40% from level ground to +9° (figure 4).

We did not find an effect of speed, slope or their interaction on the ratio of total knee positive to total individual leg positive work (*p* > 0.224, figure 4). However, there was an effect of speed (*p* = 0.001), but not slope or the interaction of speed and slope (*p* > 0.155) on the ratio of total knee negative to total individual leg negative work (figure 4). Averaged across all slopes, the ratios of total knee negative to total individual leg negative work at 1.00 and 1.25 m s^−1^ were different from 1.50 m s^−1^ (*p* < 0.001, figure 4). On level ground, the ratio of total knee positive to total individual leg positive work was 0.16 (figure 4). Averaged across all slopes, at 1.25 m s^−1^, the ratio of total knee positive to total individual leg positive work was 0.22 (figure 4). On level ground, the ratio of total knee negative to total individual leg negative work increased 18% from 1.00 to 1.50 m s^−1^ (figure 4). At 1.25 m s^−1^, the ratio of total knee negative to total individual leg negative work decreased 12% from −9° to level ground (figure 4). At 1.25 m s^−1^, the ratio of total knee negative to total individual leg negative work increased 21% from level ground to +9° (figure 4).

We found an effect of slope (*p* < 0.005) but not speed or the interaction of speed and slope on the ratio of total hip positive to total individual leg positive work (*p* > 0.495, figure 4). Averaged across all speeds, the ratios of total hip positive to total individual leg positive work at each slope were different from level ground (*p* < 0.005) except at −9° and −6° (*p* > 0.064, figure 4). We found an effect of speed and slope (*p* < 0.05), but not the interaction of speed and slope (*p* = 0.184) on the ratio of total hip negative to total individual leg negative work (figure 4). Averaged across all slopes, the ratios of total hip negative to total individual leg negative work at 1.00 and 1.25 m s^−1^ were different from 1.50 m s^−1^ (*p* < 0.001, figure 4). Averaged across all speeds, the ratios of total hip negative to total individual leg negative work at each slope were different from level ground (*p* < 0.001) except at −9°, −6° and +9° (*p* > 0.028, figure 4). On level ground, the ratio of total hip positive to total individual leg positive work was 0.33 (figure 4). At 1.25 m s^−1^, the ratio of total hip positive to total individual leg positive work decreased 9% from −9° to level ground (figure 4). At 1.25 m s^−1^, the ratio of total hip positive to total individual leg positive work increased 19% from level ground to +9° (figure 4). On level ground, the ratio of total hip negative to total individual leg negative work increased 18% from 1.00 to 1.50 m s^−1^ (figure 4). At 1.25 m s^−1^, the ratio of total hip negative to total individual leg negative work increased 6% from −9° to level ground (figure 4). At 1.25 m s^−1^, the ratio of total hip negative to total individual leg negative work increased 53% from level ground to +9° (figure 4).

### 3.4. Joint range of motion, peak moment and peak power

Ankle joint sagittal plane ROM increased with walking speed on level ground and with increasing slope (electronic supplementary material, figure S1). Knee joint sagittal plane ROM decreased on level ground with faster walking speed and with increasing slope (electronic supplementary material, figure S2). Hip joint sagittal plane ROM increased with faster walking speed on level ground and with increasing slope (electronic supplementary material, figure S3).

Ankle joint peak plantarflexion moment increased with walking speed on level ground and with increasing slope (electronic supplementary material, figure S1). Knee joint peak flexion moment and peak extension moment increased on level ground with faster walking speed and decreased with increasing slope (electronic supplementary material, figure S2). Hip joint peak flexion moment increased with faster walking speed on level ground and with increasing slope (electronic supplementary material, figure S3). Hip joint peak extension moment increased with faster walking speed on level ground and decreased with increasing slope (electronic supplementary material, figure S3).

Ankle joint peak plantarflexion power increased with walking speed on level ground and with increasing slope (electronic supplementary material, figure S1). Knee joint peak flexion power increased on level ground with faster walking speed and remained constant across slopes (electronic supplementary material, figure S2). Knee joint peak extension power decreased on level ground with faster walking speed and increased with increasing slope (electronic supplementary material, figure S2). Hip joint peak flexion power increased with faster walking speed on level ground and decreased with increasing slope (electronic supplementary material, figure S3). Hip joint peak extension power increased with faster walking speed on level ground and with increasing slope (electronic supplementary material, figure S3).

## 4. Discussion

### 4.1. Joint positive, negative and net work

Greater total positive joint and leg work with faster speed and steeper uphill slope—as well as the greater magnitude of total negative work absorbed by each joint and the individual leg with steeper downhill slope—are in support of our hypotheses and in line with previously published data [18,19]. We found similar trends (positive work increases with slope and negative work magnitude increases with downhill slope) of positive, negative and net individual leg work as those presented in Franz *et al*. [6] and Alexander *et al*. [19]. However, we found higher magnitudes of positive and negative individual leg work (1–20 times higher) than those presented in Franz *et al*. [6] and magnitudes of joint work (up to two times higher) than those presented in Alexander *et al*. [19]. These differences in magnitude are probably due to different data collection and analysis methods. We used a dual-belt instrumented treadmill to record GRFs beneath each leg at a constant speed and then used inverse dynamics to determine joint and leg sagittal plane work and power throughout the stance phase. Previous studies have used an over-ground force platform within a ramp [5,19,21] or collected GRF data from a single leg, and then used the individual limbs method to calculate COM work, and assumed symmetry between legs [6]. It is possible that by using data from both legs, averaging over many consecutive steps and using inverse dynamics to sum joint work, our data include simultaneous positive and negative work done at each joint; resulting in comparatively higher positive leg work and lower negative leg work. To compare different methods, Zelik *et al*. [7] calculated COM work via the individual limbs method, typical 3 d.f. rigid segment inverse dynamics, and a 6 d.f. ‘energy accounting’ analysis. The 6 d.f. energy accounting analysis most accurately described the work done on the COM, while the 3 d.f. and individual limbs methods underestimated the positive and negative work done at the joints, primarily at the knee and hip [7]. In the present study, we used 3 d.f. rigid segment inverse dynamics to calculate sagittal plane joint and leg work; our results corroborate the trends in joint and leg total positive and total negative work presented by previous studies [5,6,18]. Furthermore, the rigid segment inverse dynamics analyses are subject to marker tracking errors. It is possible that the increased variability in proximal segment (knee and hip) power and thus work values are due to soft tissue movement relative to the underlying bone. However, we determined the contribution from each joint relative to the overall leg, which provides insight into leg and joint function during the entire stance phase of walking over a wide range of speeds and slopes for healthy adults.

### 4.2. Joint contribution to individual leg work

Similar to Farris & Sawicki [18], we found that each joint's contribution to total positive individual leg work did not change with speed. We found that the ankle, knee and hip contributed approximately 51, 16 and 33% of total positive leg work on level ground over multiple speeds, whereas Farris & Sawicki [18] reported each joint's contribution to total positive leg power as 42, 16 and 42% for the ankle, knee and hip, respectively. There are two important differences between our study and that of Farris & Sawicki. First, we report each joint's contribution to total positive leg mechanical *work*, not total positive leg mechanical *power*. Farris & Sawicki [18] summed instantaneous joint power over the stance phase to determine total positive power. We integrated the power curve over the stance phase to calculate positive and negative joint work and summed the joint works to determine individual leg work. Joint work is important for the design of biomimetic assistive devices. Ankle work, for example, is used to modify parameters of biomimetic ankle–foot prostheses to optimize walking performance for individuals with transtibial amputations [39].

Similar to Alexander *et al*. [19], we report how joint work and each joint's contribution to individual leg work change with slope. However, we included multiple speeds at each slope, and include joint angle, moment and power. In line with Alexander *et al*. [19], we found that the ankle's contribution to individual leg positive work decreased at steeper uphill slopes (+9°) compared to level ground and that the hip's contribution to individual leg negative work increased on uphill slopes and decreased on downhill slopes compared to level ground. In contrast with Alexander *et al*. [19], we did not find a significant change in negative or positive work contribution from the knee to the individual leg on any uphill or downhill slopes. This could be attributed to differences in data collection methods. It is possible that subjects walk over a ramp at non-steady speeds, which could incur different joint work contributions. Further, absolute walking speed affects joint work. Thus, the joint work results from walking at 1.1 m s^−1^ may differ compared to the results from walking over a range of speeds. For example, a study by Donelan *et al*. [10] suggests that work done on the COM calculated via the individual limbs method for walking at 1.1 m s^−1^ is approximately 10% lower than work done on the COM for walking at 1.25 m s^−1^.

Using the combined limbs method, Winter estimated that the ankle contributed 80% of the leg work during level-ground walking [17,40]. However, Winter reported ankle and knee positive and negative work, but did not report hip, individual limb or COM work calculations. Thus, it is unclear how the estimated contribution of the ankle was calculated for level-ground walking at a steady speed and we are unable to compare our results to this reported value [17].

### 4.3. Joint range of motion

We predicted that ankle, knee and hip ROM would not change with speed or downhill slope but found that ankle ROM increased with faster speed and decreased at steeper downhill slopes, knee ROM remained constant across speeds but decreased at steeper uphill slopes and hip ROM increased with faster speed and steeper uphill slopes. In support of our prediction, and in agreement with Lay *et al*. [21], ankle ROM increased at steeper uphill slopes, as did peak plantarflexion angle. Both ankle ROM and peak ankle plantarflexion were lower at the steepest downhill slope compared to level ground (electronic supplementary material, figure S1 and table S1). As documented in Lay *et al*. [21], the ankle is mostly plantar-flexed during downhill walking and dorsi-flexed during the early and mid-stance phases of uphill walking. In accordance with data published in Lay *et al*. [21], we found peak knee flexion angle and ROM increased with steeper downhill slopes. Similarly, and in agreement with Lay *et al*. [21], we found peak hip flexion angle and ROM increased with steeper uphill slopes to accommodate leg clearance when walking uphill. There were relatively small (1–3°) differences in ROM between our results and previously published results, which is probably due to differences in marker placement and may be due to differences in over-ground versus treadmill data collection methods. Ankle and hip ROM change with both speed and slope, and knee ROM changes with slope, but each joint ROM changes more so to accommodate steeper (±9°) slopes, indicating that mechanical limitations to ROM in assistive devices may limit their function at faster walking speeds and/or when walking on steep slopes.

### 4.4. Joint moment

Our predictions regarding peak ankle, knee and hip moments were supported. Peak ankle plantarflexion moment increased with faster speed and steeper uphill slope. Peak knee joint flexion moment increased with faster speed and decreased with steeper uphill slope. Peak knee joint extension moment increased with faster speed and decreased with steeper uphill slope. Peak hip joint flexion moment increased with faster speed and with steeper uphill slope. Peak hip joint extension moment increased with faster speed and decreased with steeper uphill slope. These findings are in agreement with those documented in Lay *et al*. [21]. The external mechanical work done on the COM must increase when walking uphill and decrease when walking downhill to counteract gravity. Thus, the legs must perform more positive work on the COM when walking uphill and less positive work on the COM when walking downhill. We found that peak ankle, knee and hip moments (and thus total positive work) increase when walking uphill and decrease when walking downhill, again establishing that joint function varies when navigating slopes.

### 4.5. Joint power

Our predictions regarding peak ankle, knee and hip power were also supported. Peak ankle power increased with faster speed and steeper uphill slope to provide more positive limb power to overcome gravity when walking uphill. Peak knee joint flexion power increased with faster speed and was constant across slopes. Peak knee joint extension power remained constant with speed and slope. Peak hip joint flexion power increased with faster speed and decreased with steeper uphill slope. Peak hip joint extension power increased with faster speed and steeper uphill slope. These results are consistent with those found in Lay *et al*. [21]. The ankle provides a large portion [5,21] of the propulsive power to move the COM forward and up during the step-to-step transition, even on downhill slopes. Furthermore, and in accordance with Farris & Sawicki [18], ankle and hip power together provide 84% of individual leg positive power and 78% of individual leg positive work to walk over a range of speeds and slopes, indicating that the contribution from these joints should be considered when designing assistive devices.

### 4.6. Application to assistive device design

Understanding the function of each biological leg joint as well as their relationships to overall leg function during the stance phase of uphill and downhill walking at various speeds is critical for the design and control of robust prostheses, orthoses and other assistive devices. We found that ankle, knee and hip joint ROM, peak moment, peak power, total positive, total negative and net work change with both speed and slope. We also found that each joint's contribution to the leg changes with both speed and slope and thus should be accounted for in the control of powered assistive devices. Future studies should investigate how the addition of biomimetic ankle and/or knee power affects joint level biomechanics of people with transtibial and transfemoral amputations or people that require orthoses.

The significant dependence of ankle, knee and hip ROM, peak moment and peak power on speed and slope suggests that these should be design criteria for biomimetic assistive devices for walking to accommodate different speeds and slopes. Currently, powered ankle and knee prostheses are capable of replicating biological ankle ROM, peak moment and peak power for level-ground walking and up a 5° slope [32,33], but should be analysed at a variety of speeds and slopes to enhance the function of people with amputations or physical impairments. Powered ankle and knee prostheses are capable of generating normative work and power for a variety of speeds on level ground [23,28,29,41,42]. However, it is unknown how use of these devices affects overall walking biomechanics on uphill and downhill slopes. Powered prostheses use data input from sensors embedded in the device as well as state-space information from biological ankle work loops and knee biomechanics for level-ground walking to modulate the control of the device [23,29,30,32,33,41]. Our results show that the relationships between joint moment and angle, the relationships between joint work and leg work and the required contributions from each change significantly with walking speed and slope. The design of hardware or control systems for assistive devices that incorporate biological mechanics during uphill and downhill walking may restore normative biomechanics and reduce the metabolic cost of walking for individuals with impaired or no ankle or knee function.

## 5. Conclusion

We determined the changes in ankle, knee and hip joint biomechanics during walking at different speeds and slopes and calculated sagittal plane individual leg work and joint work over the entire stance phase. The ratio of ankle joint to total individual leg positive work did not change with speed or slope, but the ratio of ankle joint to total individual leg negative work decreased with faster speed and steeper uphill slope. The ratio of knee joint to total individual positive leg work did not change with speed or slope. However, the ratio of knee joint to total individual leg negative work increased with faster speed. The ratio of hip joint to total individual leg positive work did not change with speed but increased with steeper uphill slope. The ratio of hip joint to total individual leg negative work increased with faster speed and steeper uphill slope. Additionally, ankle and hip joint ROM, ankle, knee and hip peak moment, and peak power increased with faster speed and steeper uphill slope. In summary, walking at faster speeds requires greater positive joint work, walking up a steeper slope requires an increase in positive joint work with little increase in negative joint work and the ankle is the primary generator of this positive work, and walking down a steeper slope requires an increase in the magnitude of negative joint work with little to no increase in positive joint work and the knee is the primary absorber of this negative work.

Our results suggest that each joint facilitates the absorption and generation of work differently during level and sloped walking at various speeds. Thus, in order to replicate biological ankle, knee and hip function over a range of speeds and slopes, prosthetic or assistive devices will require dynamic (constantly changing) control of positive work production and negative work absorption as these vary within the stance phase, as well as dynamic control of peak moments, powers and ROM. Our results of the mechanical work done at each biological joint relative to the leg during walking under various conditions will inform the design, development and biomimetic control of robust prostheses, orthoses and assistive devices. The development and implementation of such devices has the potential to restore normative mechanics and metabolic costs for users and has the potential to increase quality of life [43–46], level of mobility and function [47–49], and reduce comorbidities (such as knee osteoarthritis and low back pain) commonly incurred by persons with impaired or no ankle or knee function [44,45,50].

## Supplementary Material

Supplementary Figure 1

## Supplementary Material

Supplementary Figure 2

## Supplementary Material

Supplementary Figure 3

## Supplementary Material

Supplementary Table 1

## Supplementary Material

Supplementary Table 2

## Supplementary Material

Supplementary Table 3

## Data accessibility

The authors confirm that all data underlying the findings are fully available without restriction. All relevant data are within the paper and electronic supplementary material.
