# Supplementary material for: The contributions of ankle, knee and hip joint work to individual leg work change during uphill and downhill walking over a range of speeds
Source: R Soc Open Sci. 2018 Aug 29;5(8):180550. doi: 10.1098/rsos.180550 (PMC6124028; doi:10.1098/rsos.180550)
Supplement: Supplementary Figure 3 [file rsos180550supp3.docx]

**
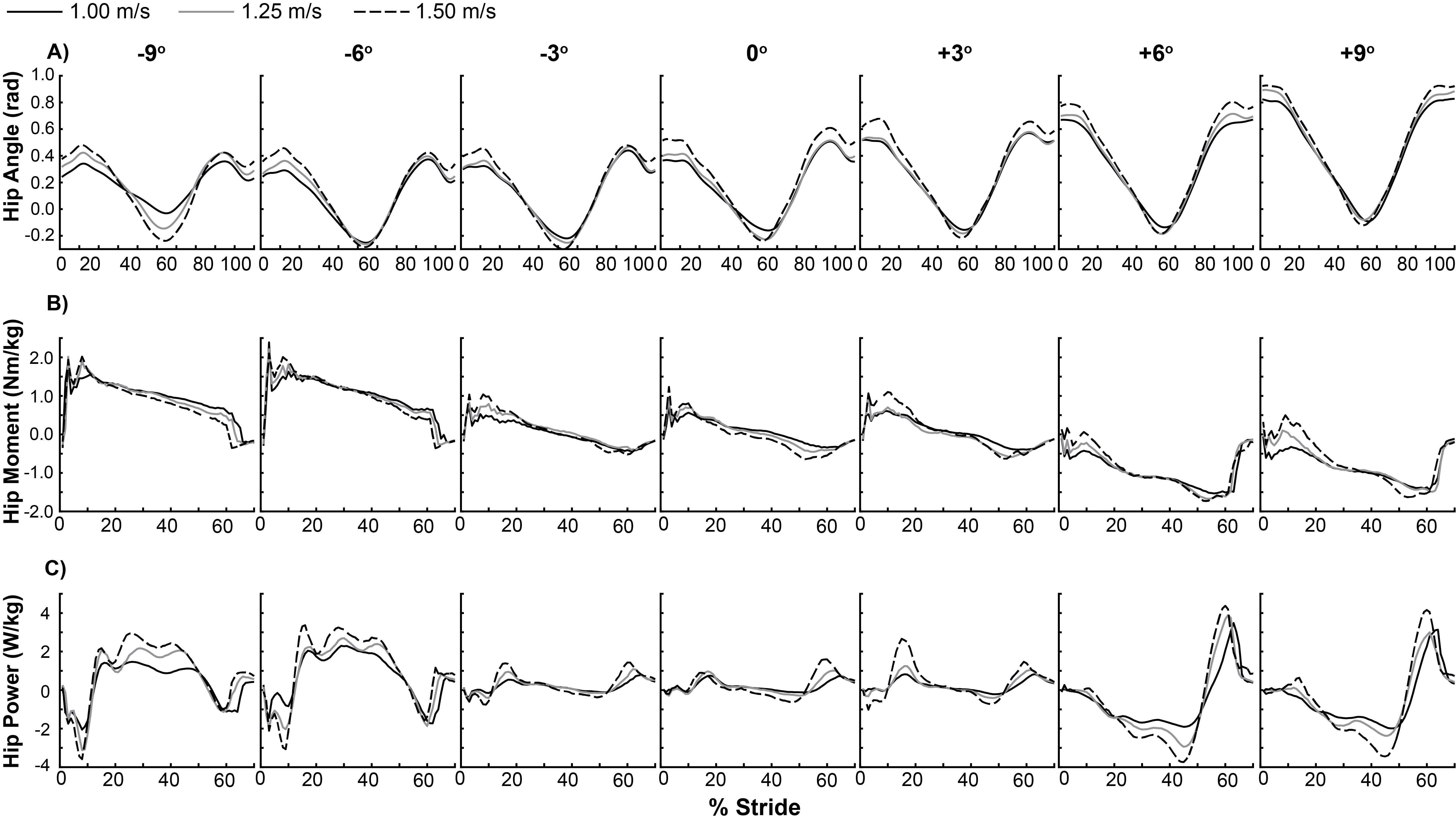
**

**Supplementary Figure 3.** Ensemble averages of 10 strides for 20 subjects’ hip joint A) angles, B) moments, and C) powers versus % of a stride starting at heel-strike for each walking speed on each slope. Hip angles are presented for the entire stride and hip joint moments and powers are presented for the stance phase only (0-70% of a stride). Each column represents a different slope from -9° (left-most column) to +9° (right-most column).
