# Supplementary material for: The contributions of ankle, knee and hip joint work to individual leg work change during uphill and downhill walking over a range of speeds
Source: R Soc Open Sci. 2018 Aug 29;5(8):180550. doi: 10.1098/rsos.180550 (PMC6124028; doi:10.1098/rsos.180550)
Supplement: Supplementary Table 1 [file rsos180550supp4.docx]

**Supplementary Table 1.** Average (S.D.) ankle joint biomechanics for all subjects walking 1.00 m/s, 1.25 m/s, and 1.50 m/s on slopes of -9° to +9°.

| **Slope (deg)** | **Speed (m/s)** | **Peak Plantarflexion Angle (rad)** | **Peak Dorsiflexion Angle (rad)** | **Range of Motion (rad)** | **Peak Moment (Nm/kg)** | **Peak Power (W/kg)** |
| --- | --- | --- | --- | --- | --- | --- |
|  |  |  |  |  |  |  |
| -9 | 1.00 | 1.14 (0.26) | 1.63 (0.26) | 0.50 (0.06) | 1.27 (0.23) | 2.84 (0.88) |
| -6 | 1.00 | 1.16 (0.24) | 1.61 (0.26) | 0.45 (0.05) | 1.35 (0.14) | 2.64 (0.72) |
| -3 | 1.00 | 1.16 (0.24) | 1.56 (0.26) | 0.40 (0.06) | 1.50 (0.19) | 2.78 (0.63) |
| 0 | 1.00 | 1.12 (0.24) | 1.54 (0.27) | 0.42 (0.07) | 1.49 (0.14) | 2.63 (0.47) |
| 3 | 1.00 | 1.07 (0.23) | 1.56 (0.26) | 0.49 (0.08) | 1.47 (0.14) | 2.73 (0.63) |
| 6 | 1.00 | 1.04 (0.27) | 1.58 (0.30) | 0.54 (0.09) | 1.52 (0.21) | 3.06 (0.65) |
| 9 | 1.00 | 1.07 (0.26) | 1.66 (0.26) | 0.59 (0.09) | 1.60 (0.24) | 3.56 (0.84) |
| -9 | 1.25 | 1.16 (0.25) | 1.63 (0.26) | 0.47 (0.07) | 1.30 (0.24) | 3.28 (1.09) |
| -6 | 1.25 | 1.14 (0.25) | 1.59 (0.27) | 0.44 (0.07) | 1.45 (0.17) | 3.36 (0.80) |
| -3 | 1.25 | 1.11 (0.24) | 1.54 (0.26) | 0.44 (0.07) | 1.63 (0.22) | 3.72 (0.81) |
| 0 | 1.25 | 1.04 (0.23) | 1.53 (0.26) | 0.49 (0.08) | 1.65 (0.14) | 3.72 (0.61) |
| 3 | 1.25 | 1.03 (0.24) | 1.58 (0.25) | 0.55 (0.08) | 1.64 (0.16) | 3.90 (0.83) |
| 6 | 1.25 | 0.99 (0.24) | 1.60 (0.25) | 0.61 (0.09) | 1.73 (0.20) | 4.34 (0.79) |
| 9 | 1.25 | 1.03 (0.26) | 1.70 (0.25) | 0.67 (0.10) | 1.81 (0.27) | 4.80 (0.96) |
| -9 | 1.50 | 1.13 (0.28) | 1.59 (0.27) | 0.46 (0.10) | 1.33 (0.25) | 3.67 (1.18) |
| -6 | 1.50 | 1.11 (0.26) | 1.56 (0.26) | 0.45 (0.08) | 1.50 (0.19) | 4.08 (0.92) |
| -3 | 1.50 | 1.03 (0.25) | 1.50 (0.27) | 0.47 (0.09) | 1.75 (0.26) | 4.65 (1.11) |
| 0 | 1.50 | 0.99 (0.23) | 1.52 (0.25) | 0.53 (0.06) | 1.81 (0.17) | 4.82 (0.74) |
| 3 | 1.50 | 0.96 (0.23) | 1.56 (0.24) | 0.60 (0.08) | 1.80 (0.20) | 4.98 (0.98) |
| 6 | 1.50 | 0.94 (0.24) | 1.59 (0.25) | 0.64 (0.09) | 1.90 (0.24) | 5.38 (0.94) |
| 9 | 1.50 | 0.94 (0.27) | 1.65 (0.28) | 0.71 (0.12) | 1.89 (0.30) | 5.59 (1.08) |
