# Supplementary material for: The contributions of ankle, knee and hip joint work to individual leg work change during uphill and downhill walking over a range of speeds
Source: R Soc Open Sci. 2018 Aug 29;5(8):180550. doi: 10.1098/rsos.180550 (PMC6124028; doi:10.1098/rsos.180550)
Supplement: Supplementary Table 2 [file rsos180550supp5.docx]

**Supplementary Table 2.** Average (S.D.) knee joint biomechanics for all subjects walking 1.00 m/s, 1.25 m/s, and 1.50 m/s on slopes of -9° to +9°.

| **Slope (deg)** | **Speed (m/s)** | **Peak Flexion Angle (rad)** | **Peak Extension Angle (rad)** | **Range of Motion (rad)** | **Peak Flexion Moment (Nm/kg)** | **Peak Flexion Power (W/kg)** | **Peak Extension Moment (Nm/kg)** | **Peak Extension Power (W/kg)** |
| --- | --- | --- | --- | --- | --- | --- | --- | --- |
|  |  |  |  |  |  |  |  |  |
| -9 | 1.00 | -1.27 (0.17) | 0.02 (0.11) | 1.29 (0.16) | -0.78 (0.36) | 0.46 (0.30) | -2.35 (1.24) | 1.30 (1.06) |
| -6 | 1.00 | -1.18 (0.14) | 0.02 (0.10) | 1.20 (0.15) | -0.56 (0.29) | 0.50 (0.38) | -1.70 (0.85) | 1.28 (1.06) |
| -3 | 1.00 | -1.03 (0.11) | 0.03 (0.09) | 1.06 (0.09) | -0.31 (0.18) | 0.60 (0.40) | -1.14 (0.39) | 1.34 (1.25) |
| 0 | 1.00 | -0.93 (0.16) | 0.01 (0.26) | 0.95 (0.16) | -0.33 (0.13) | 0.45 (0.12) | -1.00 (0.29) | 1.68 (0.28) |
| 3 | 1.00 | -0.85 (0.09) | -0.07 (0.11) | 0.79 (0.08) | -0.53 (0.39) | 0.36 (0.18) | -1.67 (1.44) | 0.73 (0.31) |
| 6 | 1.00 | -0.76 (0.12) | -0.10 (0.17) | 0.66 (0.11) | -0.69 (0.50) | 0.36 (0.18) | -1.59 (1.63) | 0.95 (0.55) |
| 9 | 1.00 | -0.75 (0.12) | -0.12 (0.18) | 0.63 (0.18) | -0.81 (0.57) | 0.41 (0.27) | -1.49 (1.47) | 1.36 (0.74) |
| -9 | 1.25 | -1.22 (0.26) | 0.07 (0.29) | 1.30 (0.10) | -1.12 (0.38) | 0.52 (0.33) | -3.49 (1.39) | 1.80 (1.18) |
| -6 | 1.25 | -1.14 (0.10) | 0.03 (0.09) | 1.17 (0.09) | -0.80 (0.32) | 0.55 (0.37) | -2.16 (0.73) | 1.66 (1.17) |
| -3 | 1.25 | -1.05 (0.13) | 0.03 (0.09) | 1.08 (0.11) | -0.55 (0.26) | 0.59 (0.39) | -1.71 (0.49) | 1.56 (1.16) |
| 0 | 1.25 | -0.95 (0.09) | 0.03 (0.07) | 0.97 (0.08) | -0.50 (0.15) | 0.51 (0.14) | -1.34 (0.33) | 1.10 (0.39) |
| 3 | 1.25 | -0.88 (0.10) | -0.06 (0.10) | 0.82 (0.08) | -0.70 (0.42) | 0.44 (0.21) | -2.21 (1.65) | 1.19 (0.49) |
| 6 | 1.25 | -0.80 (0.11) | -0.09 (0.17) | 0.71 (0.12) | -0.84 (0.52) | 0.43 (0.20) | -2.05 (1.83) | 1.35 (0.70) |
| 9 | 1.25 | -0.76 (0.12) | -0.07 (0.21) | 0.70 (0.18) | -1.00 (0.58) | 0.51 (0.29) | -1.89 (1.65) | 1.85 (0.89) |
| -9 | 1.50 | -1.17 (0.21) | 0.07 (0.29) | 1.24 (0.13) | -1.33 (0.37) | 0.57 (0.29) | -4.69 (1.58) | 2.39 (1.38) |
| -6 | 1.50 | -1.05 (0.16) | 0.04 (0.14) | 1.09 (0.14) | -1.01 (0.28) | 0.52 (0.33) | -3.04 (0.97) | 1.89 (1.22) |
| -3 | 1.50 | -0.96 (0.20) | 0.08 (0.31) | 1.04 (0.20) | -0.60 (0.32) | 0.72 (0.39) | -1.98 (0.82) | 2.24 (1.25) |
| 0 | 1.50 | -0.92 (0.08) | 0.01 (0.08) | 0.93 (0.07) | -0.69 (0.18) | 0.57 (0.15) | -1.73 (0.35) | 1.44 (0.50) |
| 3 | 1.50 | -0.85 (0.11) | -0.06 (0.10) | 0.79 (0.09) | -0.83 (0.43) | 0.50 (0.24) | -2.59 (1.77) | 1.61 (0.60) |
| 6 | 1.50 | -0.80 (0.09) | -0.04 (0.22) | 0.76 (0.21) | -0.92 (0.51) | 0.53 (0.23) | -2.98 (3.08) | 2.18 (1.96) |
| 9 | 1.50 | -0.76 (0.13) | -0.03 (0.28) | 0.73 (0.22) | -1.06 (0.62) | 0.57 (0.30) | -2.43 (1.96) | 2.38 (1.34) |
