# Supplementary material for: The contributions of ankle, knee and hip joint work to individual leg work change during uphill and downhill walking over a range of speeds
Source: R Soc Open Sci. 2018 Aug 29;5(8):180550. doi: 10.1098/rsos.180550 (PMC6124028; doi:10.1098/rsos.180550)
Supplement: Supplementary Table 3 [file rsos180550supp6.docx]

**Supplementary Table 3.** Average (S.D.) hip joint biomechanics for all subjects walking 1.00 m/s, 1.25 m/s, and 1.50 m/s on slopes of -9° to +9°.

| **Slope (deg)** | **Speed (m/s)** | **Peak Flexion Angle (rad)** | **Peak Extension Angle (rad)** | **Range of Motion (rad)** | **Peak Flexion Moment (Nm/kg)** | **Peak Flexion Power (W/kg)** | **Peak Extension Moment (Nm/kg)** | **Peak Extension Power (W/kg)** |
| --- | --- | --- | --- | --- | --- | --- | --- | --- |
|  |  |  |  |  |  |  |  |  |
| -9 | 1.00 | -0.14 (0.13) | 0.21 (0.13) | 0.36 (0.06) | -0.56 (0.22) | 0.99 (0.51) | -0.77 (0.51) | 0.98 (0.38) |
| -6 | 1.00 | -0.22 (0.14) | 0.22 (0.13) | 0.44 (0.06) | -0.47 (0.18) | 1.02 (0.63) | -0.69 (0.70) | 1.01 (0.43) |
| -3 | 1.00 | -0.26 (0.14) | 0.24 (0.13) | 0.50 (0.10) | -0.42 (0.16) | 1.04 (0.54) | -0.70 (0.85) | 1.15 (0.62) |
| 0 | 1.00 | -0.28 (0.12) | 0.28 (0.12) | 0.56 (0.11) | -0.51 (0.17) | 0.83 (0.25) | -0.29 (0.27) | 0.86 (0.21) |
| 3 | 1.00 | -0.26 (0.13) | 0.41 (0.15) | 0.67 (0.09) | -0.75 (0.51) | 0.70 (0.40) | -0.56 (0.62) | 1.50 (0.90) |
| 6 | 1.00 | -0.25 (0.11) | 0.58 (0.13) | 0.83 (0.08) | -0.77 (0.60) | 0.64 (0.41) | -0.72 (0.89) | 1.86 (1.17) |
| 9 | 1.00 | -0.22 (0.11) | 0.74 (0.14) | 0.97 (0.12) | -0.71 (0.55) | 0.78 (0.46) | -0.87 (1.46) | 2.28 (1.01) |
| -9 | 1.25 | -0.24 (0.11) | 0.25 (0.10) | 0.49 (0.09) | -0.66 (0.27) | 1.28 (0.53) | -1.35 (0.64) | 1.35 (0.47) |
| -6 | 1.25 | -0.29 (0.13) | 0.27 (0.11) | 0.56 (0.08) | -0.60 (0.21) | 1.23 (0.60) | -1.00 (0.63) | 1.47 (0.63) |
| -3 | 1.25 | -0.27 (0.14) | 0.34 (0.13) | 0.60 (0.08) | -0.58 (0.21) | 1.16 (0.54) | -0.86 (0.74) | 1.54 (0.77) |
| 0 | 1.25 | -0.32 (0.11) | 0.35 (0.12) | 0.67 (0.07) | -0.68 (0.27) | 1.01 (0.24) | -0.49 (0.36) | 1.24 (0.34) |
| 3 | 1.25 | -0.29 (0.13) | 0.47 (0.13) | 0.76 (0.10) | -0.98 (0.51) | 0.91 (0.51) | -0.99 (0.90) | 2.25 (1.71) |
| 6 | 1.25 | -0.28 (0.11) | 0.67 (0.14) | 0.95 (0.08) | -0.97 (0.62) | 0.92 (0.51) | 1.01 (1.12) | 2.48 (1.15) |
| 9 | 1.25 | -0.26 (0.11) | 0.84 (0.14) | 1.10 (0.11) | -0.86 (0.56) | 1.04 (0.53) | -0.90 (0.99) | 2.71 (0.84) |
| -9 | 1.50 | -0.28 (0.13) | 0.33 (0.13) | 0.61 (0.09) | -0.76 (0.31) | 1.60 (0.53) | 2.05 (0.89) | 1.89 (0.77) |
| -6 | 1.50 | -0.33 (0.13) | 0.34 (0.13) | 0.67 (0.08) | -0.72 (0.23) | 1.43 (0.52) | 1.31 (0.67) | 1.74 (0.61) |
| -3 | 1.50 | -0.35 (0.14) | 0.37 (0.13) | 0.72 (0.09) | -0.66 (0.27) | 1.56 (0.54) | 1.04 (0.72) | 2.11 (0.92) |
| 0 | 1.50 | -0.36 (0.12) | 0.43 (0.12) | 0.79 (0.08) | -0.83 (0.24) | 1.30 (0.28) | 1.08 (1.85) | 1.68 (0.48) |
| 3 | 1.50 | -0.31 (0.13) | 0.56 (0.14) | 0.87 (0.11) | -1.09 (0.53) | 1.17 (0.62) | 1.17 (0.83) | 2.44 (1.07) |
| 6 | 1.50 | -0.31 (0.11) | 0.73 (0.13) | 1.04 (0.08) | -1.14 (0.59) | 1.29 (0.61) | 1.45 (1.35) | 3.31 (1.18) |
| 9 | 1.50 | -0.26 (0.12) | 0.91 (0.14) | 1.17 (0.12) | -1.07 (0.61) | 1.34 (0.66) | 1.33 (1.25) | 3.51 (1.03) |
